# In a porcine model of implantable pacemakers for pediatric unilateral diaphragm paralysis, the phrenic nerve is the best target

**DOI:** 10.1186/s13019-024-02707-w

**Published:** 2024-04-05

**Authors:** Tobias Kratz, Jan Dauvergne, Roman Ruff, Timo Koch, Johannes Breuer, Boulos Asfour, Ulrike Herberg, Benjamin Bierbach

**Affiliations:** 1 Department of Paediatric Cardiology, University Hospital Bonn, Venusberg-Campus 1, 53127 Bonn, Germany; 2 Fraunhofer IBMT, Institute for Biomedical Engineering, Sulzbach, Germany; 3 Department of Pediatric Cardiac Surgery, University Hospital Bonn, Bonn, Germany; 4 Department of Pediatric Cardiology, University Hospital Aachen, Aachen, Germany

**Keywords:** Unilateral diaphragmatic paralysis, Diaphragm pacemaker, Fontan circulation, Univentricular heart, Stimulation

## Abstract

**Background:**

A frequent complication of Fontan operations is unilateral diaphragmatic paresis, which leads to hemodynamic deterioration of the Fontan circulation. A potential new therapeutic option is the unilateral diaphragmatic pacemaker. In this study, we investigated the most effective stimulation location for a potential fully implantable system in a porcine model.

**Methods:**

Five pigs (20.8 ± 0.95 kg) underwent implantation of a customized cuff electrode placed around the right phrenic nerve. A bipolar myocardial pacing electrode was sutured adjacent to the motor point and peripherally at the costophrenic angle (peripheral diaphragmatic muscle). The electrodes were stimulated 30 times per minute with a pulse duration of 200 µs and a stimulation time of 300 ms. Current intensity was the only variable changed during the experiment.

**Results:**

Effective stimulation occurred at 0.26 ± 0.024 mA at the phrenic nerve and 7 ± 1.22 mA at the motor point, a significant difference in amperage (*p* = 0.005). Even with a maximum stimulation of 10 mA at the peripheral diaphragm muscle, however, no effective stimulation was observed.

**Conclusion:**

The phrenic nerve seems to be the best location for direct stimulation by a unilateral thoracic diaphragm pacemaker in terms of the required amperage level in a porcine model.

## Introduction

The diaphragmatic pacemaker is a therapeutic option for the treatment of diaphragmatic paresis and used for bilateral paresis or dysfunction. This device is indicated for patients with high-level spinal cord injuries or central hypoventilation syndrome [1]. Two versions of the pacemaker are in use, one that provides direct bilateral stimulation of the phrenic nerve [2, 3] and one that provides bilateral stimulation of the abdominal motor point [4]. Both systems stimulate the diaphragm via external stimulation without triggering. A fully implantable pacemaker for unilateral diaphragmatic paresis that stimulates the paretic side by triggering the healthy side is not currently available, however. Unilateral diaphragmatic paresis is a known complication with Fontan surgery [5–9] and has an important influence on outcomes [10, 11].

The Fontan pathway is a multi-stage treatment performed on children with univentricular circulation. With the connection of the superior and inferior venae cavae to the pulmonary arteries, the deoxygenated blood flows passively into the pulmonary arteries without a sub-pulmonary ventricle [12]. Negative intrathoracic pressure is an important factor supporting passive pulmonary blood flow and is generated within the closed chest by diaphragmatic excursion. The importance of spontaneous breathing in patients with Fontan circulation was first demonstrated by Redington and Penny. Their report that intrathoracic negative pressure generated in the lungs during inspiration increases antegrade blood flow in the pulmonary arteries [13, 14] was later confirmed in MRI studies [15, 16].

Usually, symptomatic Fontan patients with diaphragmatic palsy are treated with diaphragmatic plication, which unfortunately does not yield hemodynamic improvement in the Fontan circulation [17]. Diaphragmatic plication thus does not seem to be an optimal treatment, and its effect on improving pulmonary blood flow has not been consistently demonstrated, especially in patients with univentricular heart and diaphragmatic palsy.

We are developing a triggered unilateral diaphragmatic pacing system for use as a pacemaker, especially in children after Fontan surgery. Our goal is to improve the reduced pulmonary blood flow in the paretic side by diaphragmatic pacing.

For such a pacemaker, an accelerometer may be utilized as a potential device for triggering [18]. Based on our animal model [19], in the current experiment, we are going to identify which stimulation location is the best target for a unilateral diaphragm pacemaker in our porcine model. For this purpose we compared the peripheral diaphragm muscle, phrenic nerve, and nerve entry point into the diaphragm (also called the motor point [20]) in terms of the amperage required to generate sufficient stimulation. Additionally, we performed the experiment with a transected phrenic nerve to exclude the influence of spontaneous breathing.

Although several stimulus thresholds have already been determined in humans [1, 21, 22] and mongrel dogs [23, 24] an evaluation in the porcine model does not exist yet. Although the porcine model represents one of the most frequently used large animal models in Europe at present [25]. Hence, it seems to be of major interest to implement such an examination in order to demonstrate the transferability of our porcine model’s data to human patients.

## Material and methods

Experiments were conducted in accordance with the German Animal Welfare Act and its subsequent statutory acts, which are in accordance with the Council of Europe Convention ETS 123. The competent state agency, the State Office for Nature, Environment and Consumer Protection North Rhine-Westphalia, approved the study (permit: 81–02.04.2020.A392). Our study complied with the Animal Research: Reporting of In Vivo Experiments guidelines 2.0.

### Animal preparation and instrumentation

Five pigs were examined and had a mean weight of 20.8 ± 0.95 kg. Pigs were supplied in-house by the Agriculture Faculty, Rheinische Friedrich-Wilhelms-University Bonn, Königswinter-Vinxel, Germany, were of conventional microbiologic status, and had an acclimatization period of 3 days at our facility.

Premedication consisted of intramuscularly applied ketamine (20 mg/kg; WDT, Garbsen, Germany) in combination with azaperone (2 mg/kg; Richter Pharma, Wels, Austria) and atropine (0.02 mg/kg; B. Braun, Melsungen, Germany). After adequate sedation was achieved, venous access was implemented with a 1.1 mm outer diameter Jelco® catheter (Smith Medical, Grasbrunn, Germany) in an ear vein. Anesthesia induction consisted of piritramide (0.5 mg/kg; Hameln Pharma, Hameln, Germany) and propofol (10 mg/kg; CP Pharma, Burgdorf, Germany). The administration of muscle relaxants was explicitly avoided in this experiment. We secured the airway via endotracheal intubation using a straight size 4 Miller blade with a 4.5 mm internal diameter curved, microcuffed endotracheal tube (Avanos, Hamburg, Germany). For invasive ventilation, we used a Servo-i (Maquet, Rastatt, Germany) with synchronized intermittent mandatory ventilation with a frequency of around 15 breaths per minute, an inspiratory pressure of 15 cmH_2_O, and a positive end-expiratory pressure of 5 cmH_2_O. The animals were ventilated on room air (F_i_O_2_ = 0.21). We used a flow trigger mode set to > 1 l/min. Anesthesia was maintained with continuous intravenous infusion of propofol (1–5 mg/kg/h) via a Perfusor® Space (B. Braun, Melsungen, Germany) and continuous intravenous infusion of piritramide (0.2–0.5 mg/kg), supplemented by occasional single doses of ketamine (5–10 mg/kg/h) or midazolam (0.5 mg/kg; B. Braun, Melsungen, Germany). We monitored the depth of total intravenous anesthesia with the Narcotrend system via a Compact M monitor for intraoperative use (Narcotrend, Hannover, Germany) using needle electrodes (Neuroline Twisted Pair Subdermal, 12 × 0.4 mm, Ambu, Ballerup, Denmark) placed at the standard positions according to the manufacturer’s instructions. During the procedure, the animals were in electroencephalographic Kugler stage D0 [26], which is equivalent to general anesthesia.

We monitored the pigs using an Infinity C500 monitor (Dräger, Lübeck, Germany) for electrocardiogram, pulse oximetry, and invasive blood pressure. For capnometry, we used a Datex-Ohmeda S/5 (Datex-Ohmeda, Duisburg, Germany). We placed an arterial line (2.7 French leadercath, Vygon, Aachen, Germany) in the right femoral artery for continuous blood pressure monitoring and a three-lumen central line (5.5 French, Teleflex, Fellbach, Germany) in the right femoral vein. In addition, we placed a 10 Charrière transurethral catheter (Asid Bonz, Herrenberg, Germany) to monitor urine output. Temperature was measured with a 9 French rectal probe (Smiths Medical, Grasbrunn, Germany). Maintenance fluids were infused at a rate of 50–70 mL/kg/h using Ionosteril 1/1 (Fresenius Kabi, Bad Homburg, Germany). At the end of the experiment, animals were euthanized using T61® (tetracaine/mebezonium/embutramide) (Intervet, München, Germany) at a dose of 0.5 mL/kg.

### Intervention set-up

**Fig. 1:**
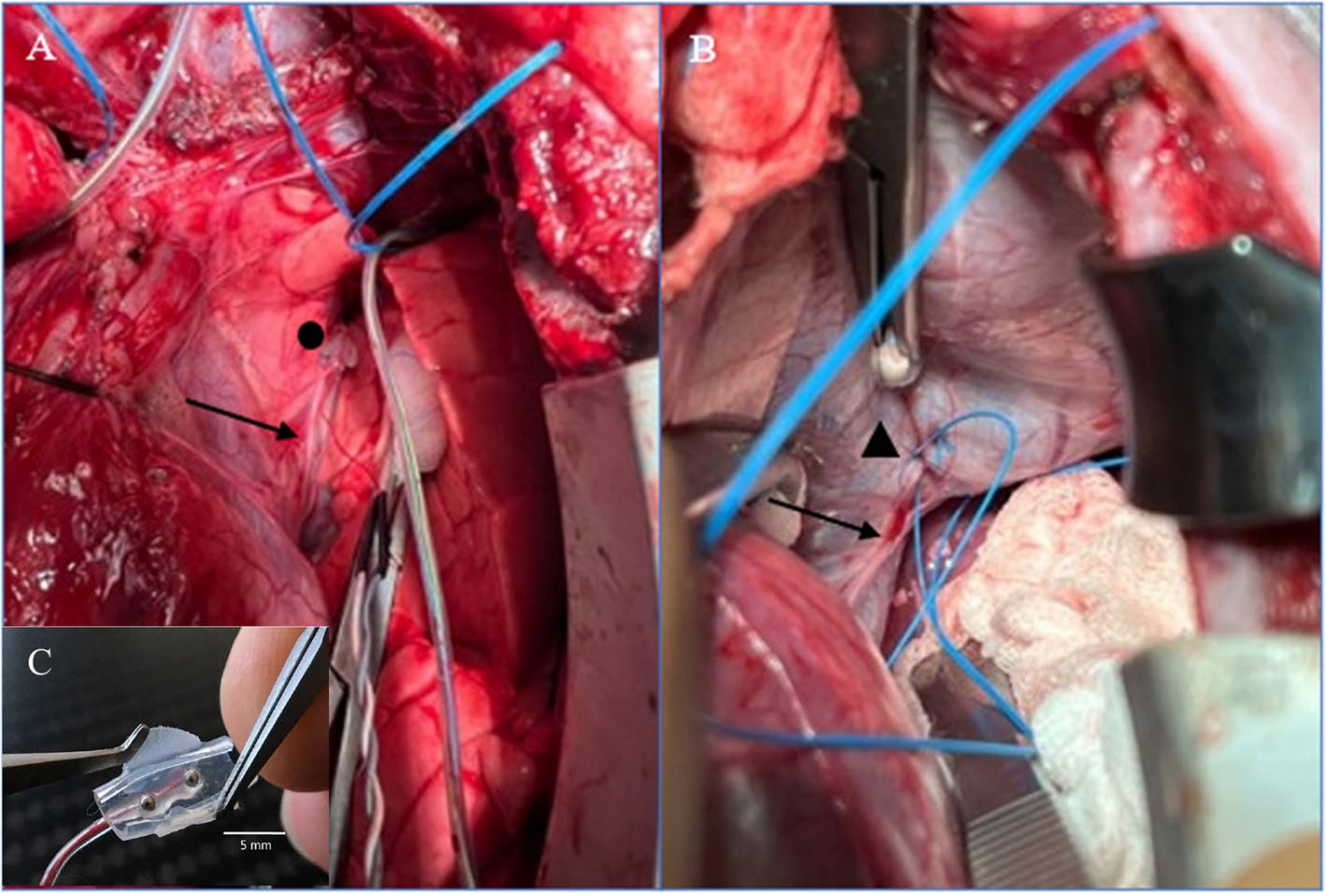
**A** Cuff electrode placement around the phrenic nerve (●). **B** Bipolar TME electrode implantation at the motor point (▲); → Indicates the phrenic nerve. **C** Cuff electrode

After initial supine positioning, the pigs were prepared and instrumented as described above. A median sternotomy was performed, the pleura opened, and the right diaphragm exposed, along with the phrenic nerve in continuation. The electrodes were then implanted using 5–0 polypropylene sutures (Ethicon, Norderstedt, Germany). A custom-made bipolar cuff electrode built by the Fraunhofer Institute for Biomedical Engineering (Sulzbach, Germany) was placed around the right phrenic nerve (Fig. 1A). A bipolar myocardial pacing electrode, TME (Osypka, Rheinfeld, Germany), was implanted adjacent to the motor point (Fig. 1B) and peripherally on the diaphragmatic muscle at the costo-diaphragmatic angle. The motor point was identified visually. The electrodes were sutured on each side of the nerve’s entry point into the diaphragm. The two poles of the electrode each were implanted at a distance of 1 cm. After the instrumentation the animals were allowed to recover for a period of 30 to 45 min. In this recovery period a bolus (10 ml/ kg bodyweight) of Ionosteril 1/1 (Fresenius Kabi, Bad Homburg, Germany) was administered closely observing the central venous pressure (target: 5 to 10 mmHg). Ventilation was adjusted in order to provide a balanced acid–base state. The sternal retractor was removed, and the chest was left open. The incision was covered by a moisted swap.

The electrodes were then stimulated 30 times per minute with a pulse duration of 200 µs and a stimulation time of 300 ms. Only current intensity was varied. The stimulation was carried out using a FE180 (ADInstruments, Sydney, Australia) and ISIS Neurostimulator (inomed, Emmendingen, Germany). The stimulation unit was placed on a separate mount extracorporeally. For each amperage, stimulation was performed for at least for 5 min.

Stimulation was conducted with 0.1, 0.2, 0.3, and 0.5 mA at the phrenic nerve, with 0.1, 1, 3, 5, and 10 mA at the motor point, and with 5 and 10 mA at the peripheral diaphragm muscle. The recording of lower stimulation currents at the peripheral diaphragm muscle was not performed, as no effective stimulation could be achieved. The same series of stimulations was carried out after the phrenic nerve had been transected approximately 2 cm above the cuff electrode.

### Measurement

All measurements were taken at baseline without phrenic nerve stimulation with the intact phrenic nerve and at the different stimulation levels still with the phrenic nerve being intact. Thereafter, the phrenic nerve was transected, and the same varying stimulation levels were applied. Diaphragmatic movement was recorded by fluoroscopy (Ziehm Vista, Ziehm, Nuremberg, Germany) and evaluated with a tangent drawn at the highest point of the right hemi-diaphragm. At this point, diaphragmatic movement was measured at a right angle in millimeters. Arterial blood gas analyses were conducted in a RapidPoint® 500 analyzer (Siemens Healthineers, Erlangen, Germany). Ventilation parameters were measured using Servo-i (Maquet, Rastatt, Germany), and baseline ventilation settings were an inspiratory pressure of 15 cmH_2_O, a positive end-expiratory pressure of 5 cmH_2_O, and a ventilation rate of 15/min.

### Statistics

Data are presented as mean ± SEM. The statistical significance of changes from baseline values within each parameter was tested with ANOVA for repeated measures. Differences between the various stimulation locations were analyzed by one-way ANOVA comparing the three stimulation locations.

Diaphragmatic excursion was defined as complete downward motion of the entire diaphragmatic plane. The threshold was detected at the three locations, and threshold mean values from all animals at the individual locations were compared for identifying the best location for diaphragmatic stimulation. Normal distribution was tested by the Shapiro–Wilk test. For nonparametric values, the Kruskal–Wallis test was used. Differences between the intact and the transected states were compared with independent t-tests. Statistical significance was accepted at *p* ≤ 0.05 after pairwise testing. Statistical analyses were performed with IBM SPSS Statistics Version 28.

## Results

At baseline, mean values were as follows: pH, 7.44 ± 0.02; respiratory minute volume, 2.76 ± 0.18 l/min; pO_2_, 71.8 ± 2.01 mmHg; end-tidal CO_2_, 38.2 ± 0.97 mmHg; breathing rate, 15/min; and diaphragmatic excursion, 4.2 ± 0.3 mm.

### Stimulation

#### Phrenic nerve

**Fig. 2:**
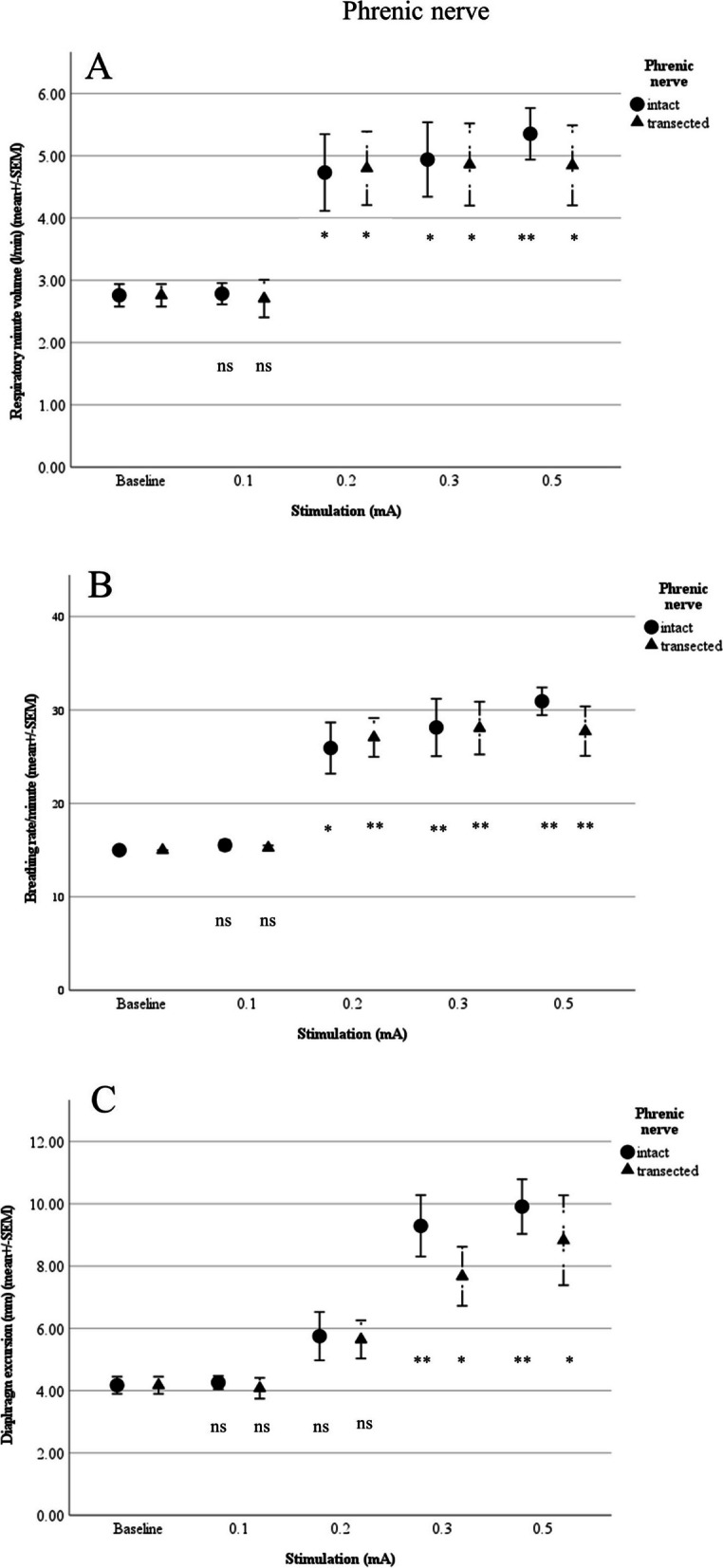
**A** Respiratory minute volume (L/min), **B** Breathing rate (per min) and **C** Diaphragm excursion in relation to stimulation of the phrenic nevre (*n* = 5; ns: not siginificant; *p<0.05, **p<0.01, ***p<0.001)

**Fig. 3:**
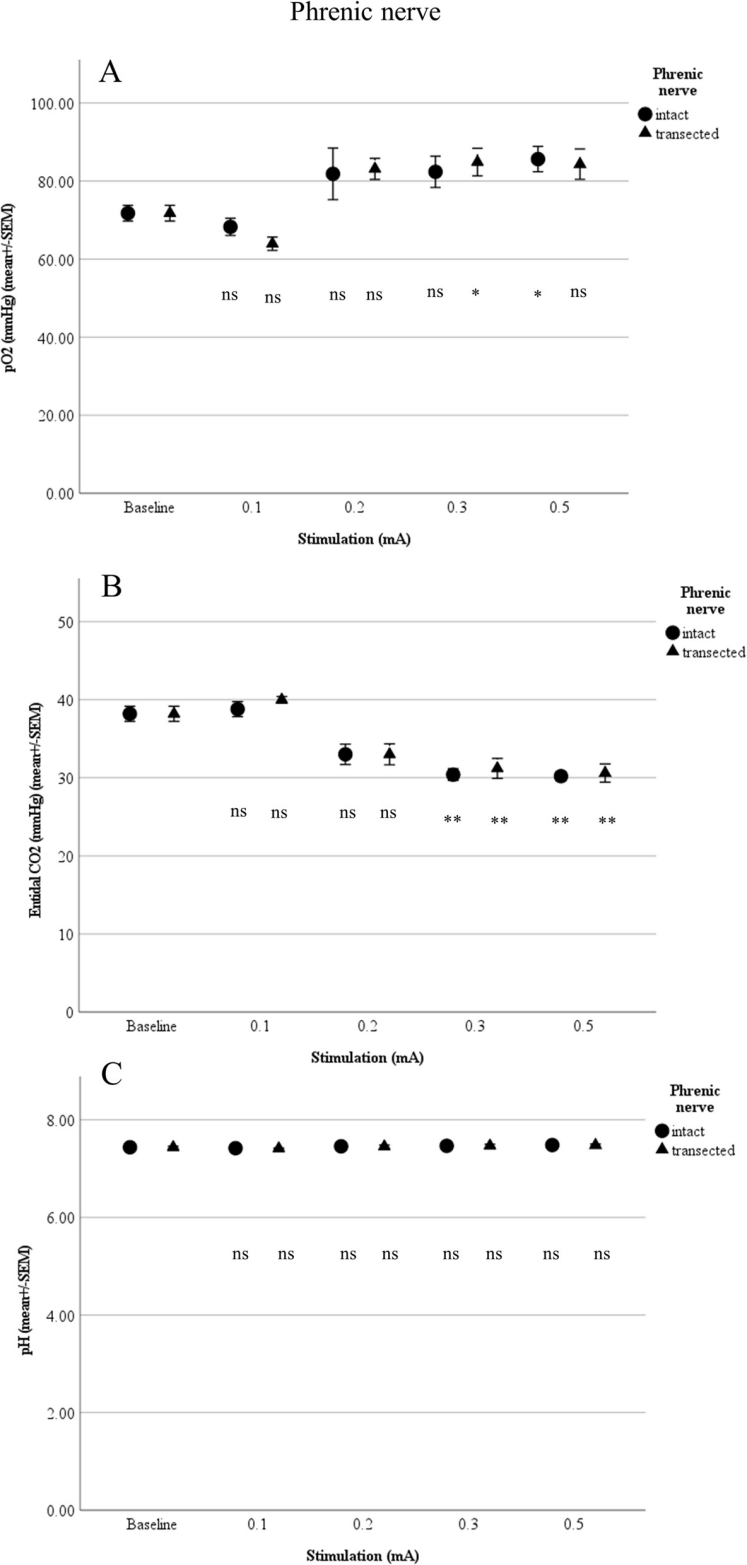
**A** pO_2_, **B** End-tidal CO_2_, and **C** pH in relation to stimulation of the phrenice nerve (*n* =5; ns:not significant; *p <0.05, **p <0.01, ***p <0.001)

Compared with the above baseline values, direct stimulation at a threshold of 0.2 mA resulted in significantly increased respiratory minute volume to 4.73 ± 0.62 L/min (*p* = 0.019) and breathing rate to 25.93 ± 2.73/min (*p* = 0.05). At 0.3 mA, compared with baseline, there were significant changes in end-tidal CO_2_ (to 30.4 ± 0.75 mmHg; *p* = 0.016) and diaphragm excursion (to 9.3 ± 1 mm; *p* = 0.003). Change from baseline pO_2_ was not seen until application of 0.5 mA (to 85.64 ± 3.24 mmHg; *p* = 0.039), and no change in pH was detected at any level (Figs. 2 and 3).

After the right phrenic nerve was transected, compared with baseline, there were significant changes in respiratory minute volume (4.86 ± 0.66 L/min; *p* = 0.024) and breathing rate (27.07 ± 2.08/min; *p* = 0.02) at 0.2 mA. At 0.3 mA, changes from baseline were seen for end-tidal CO_2_ (31.2 ± 1.28 mmHg; *p* = 0.003), diaphragm excursion (7.7 ± 0.95 mm; *p* = 0.032), and pO_2_ (84.88 ± 3.53 mmHg; *p* = 0.042). Values for pH remained unchanged (Figs. 2 and 3).

We found no significant difference in mean values between intact and transected phrenic nerves (Figs. 2 and 3).

#### Motor point

**Fig. 4:**
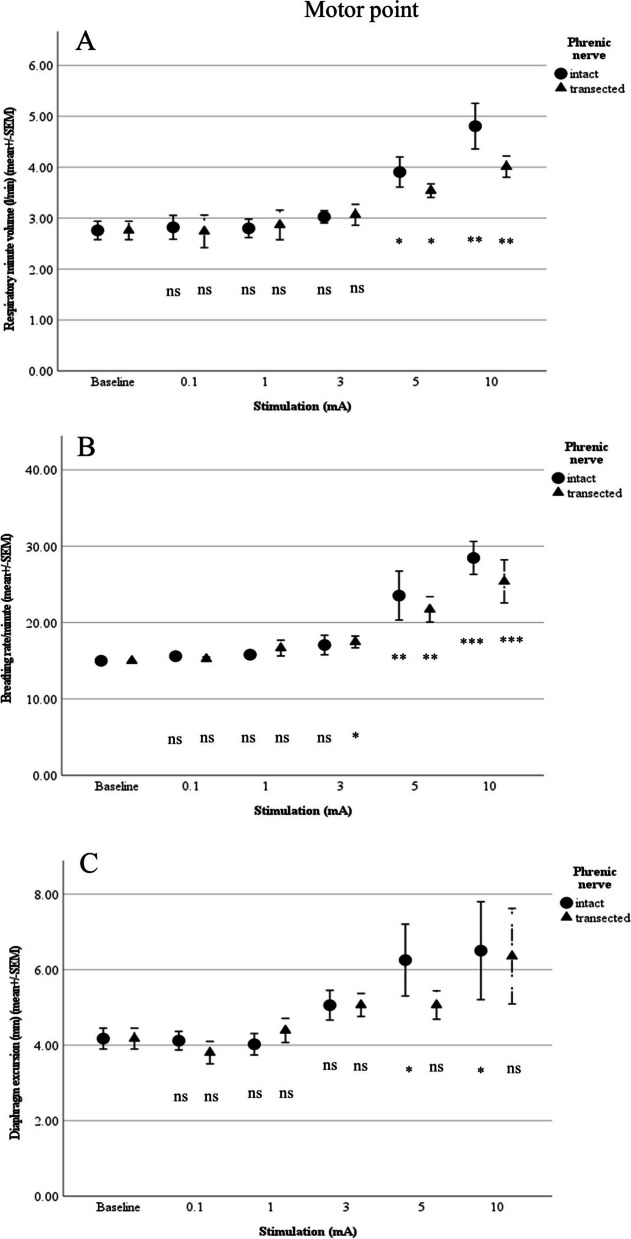
**A** Respiratory minute volume (L/min), **B** Breathing rate (per min) and **C** Diaphragm excursion in relation to stimulation of the motor point (*n*= 5; ns: not significant; *p<0.05, **p<0.01, ***p<0.001)

**Fig. 5:**
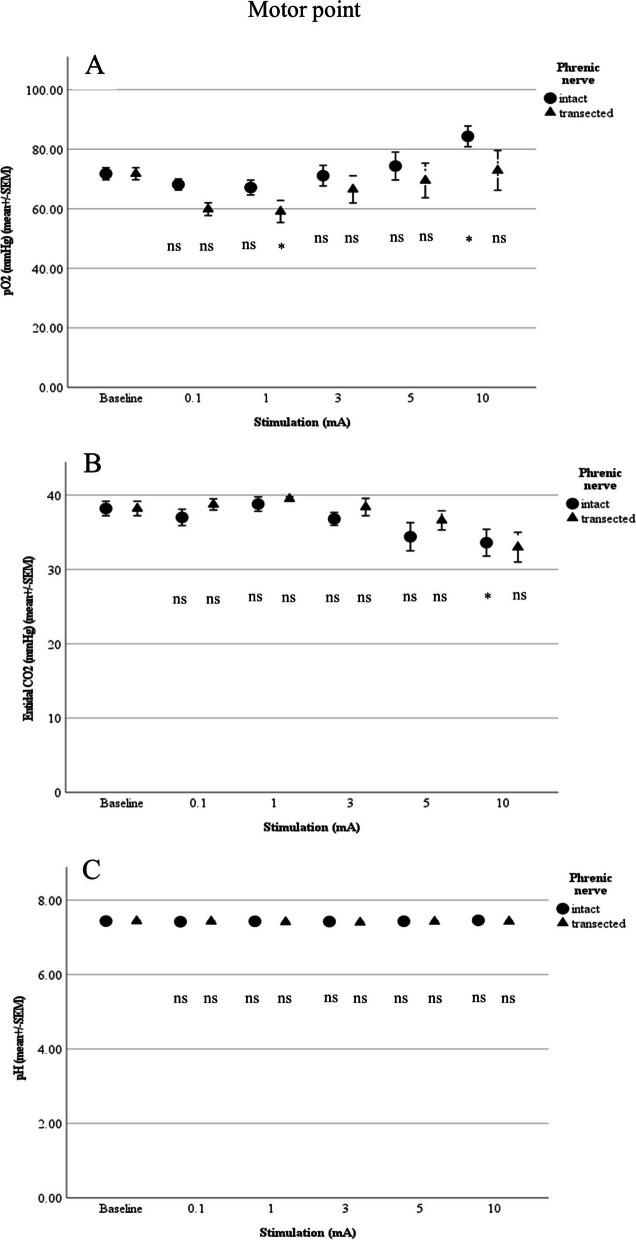
**A** pO_2_, **B** End-tidal CO_2_ and **C** pH in relation to stimulation of the motor point (*n*= 5; ns: not significant; *p<0.05, **p<0.01, ***p<0.001)

Motor point stimulation with the phrenic nerve intact differed significantly from baseline at 5 mA for respiratory minute volume (3.9 ± 0.30 L/min; *p* = 0.017), breathing rate (23.5 ± 3.2/min; *p* = 0.009), and diaphragm excursion (6.3 ± 1 mm; *p* = 0.01). Significant changes from baseline were observed for end-tidal CO_2_ (33.6 ± 1.81 mmHg; *p* = 0.046) and pO_2_ (84.3 ± 3.48 mmHg; *p* = 0.045) with 10 mA. No significant difference could be seen for pH as a function of the different amperage levels (Figs. 4 and 5).

After transection of the phrenic nerve, stimulation of the motor point yielded no difference from baseline for end-tidal CO_2_, pO_2_, pH, or diaphragm excursion. For breathing rate, a significant change was noted at 3 mA (to 17.46 ± 0.76/min; *p* = 0.026), as was also the case for respiratory minute volume (to 3.5 ± 0.13 L/min; *p* = 0.044) at 5 mA.

Mean values between intact and transected nerves during stimulation of the motor point differed no significant difference could be observed between the two groups (Figs. 4 and 5).

#### Peripheral diaphragm muscle

Although a local contraction in the vicinity of the electrodes could be visually perceived, complete diaphragmatic contraction was not achieved. However, maximum stimulation (10 mA) of the peripheral diaphragmatic muscle with the nerve intact yielded a significant change in breathing rate (18.53 ± 3.57/min; *p* = 0.026) and diaphragm excursion (5.32 ± 0.50 mm; *p* = 0.028). After transection of the nerve, there was a significant change in breathing rate only with 10 mA (20 ± 5.9/min; *p* = 0.019). At 5 mA, we also observed a significant deterioration in pO_2_ (60.62 ± 8.13 mmHg; *p* = 0.027) from baseline (71.8. ± 4.5 mmHg).

**Fig. 6:**
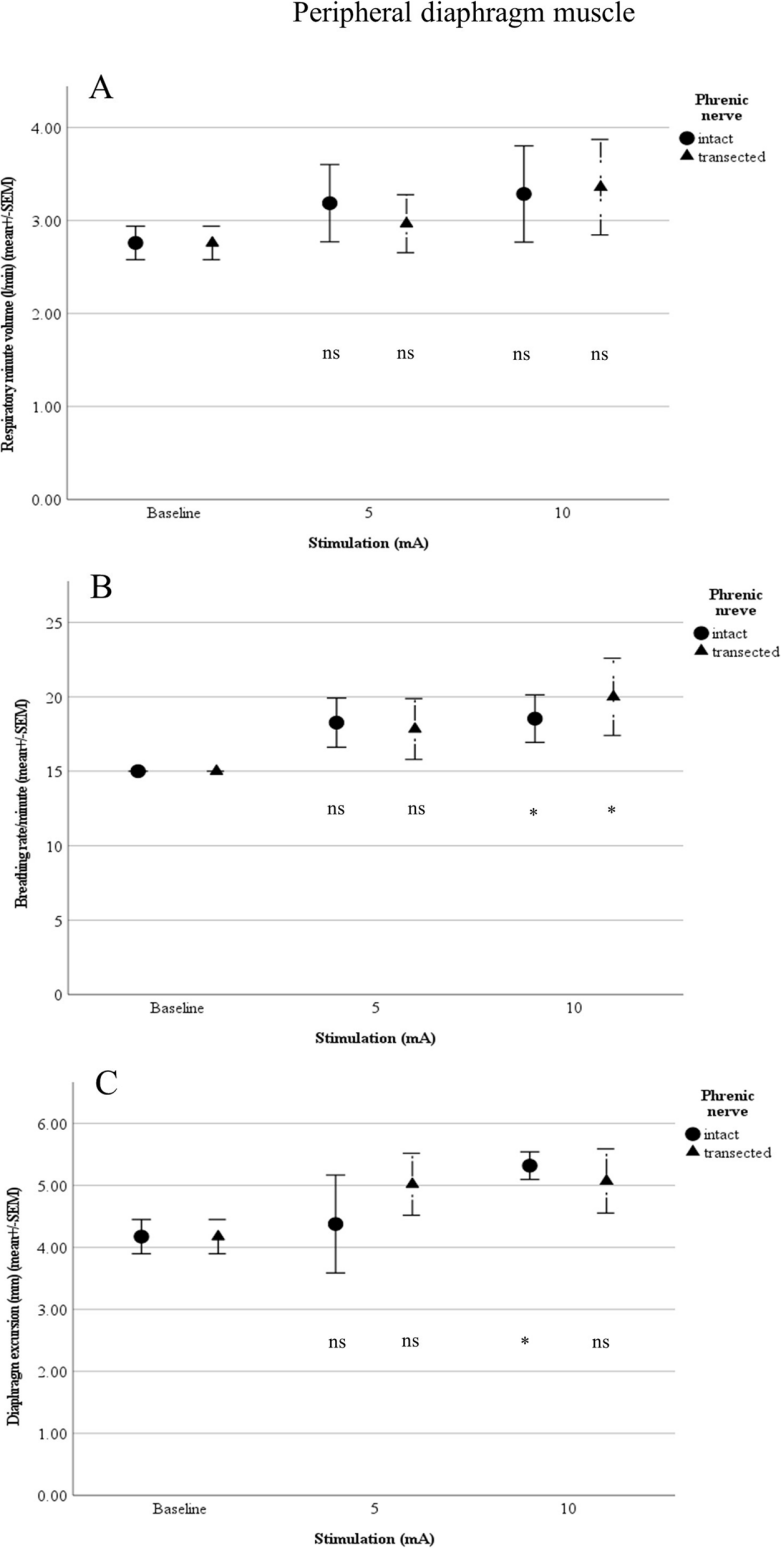
**A** Respiratory minute volume (L/min), **B** Breathing rate (per min) and **C** Diaphragm excursion in relation to stimulation of the peripheral diaphragm muscle (*n*=5; ns:not significant; *p<0.05, **p<0.01, ***p<0.001)

**Fig. 7:**
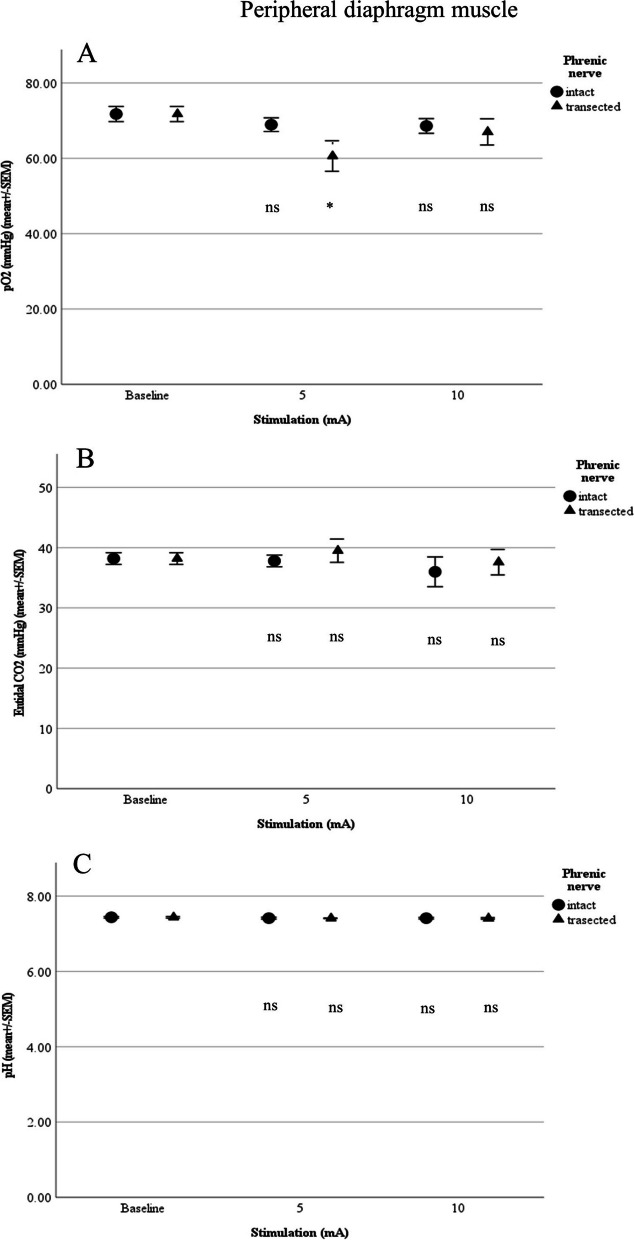
**A** pO_2_, **B** End-tidal CO_2_ and **C **pH in relation to stimulation of the peripheral diaphragm muscle (*n*=5; ns:not significant; *p<0.05, **p<0.01, ***p<0.001)

Comparison of mean values at the peripheral diaphragm muscle with the intact and transected phrenic nerve showed no significant difference (Figs. 6 and 7).

### Comparison of the three stimulation locations

The comparison between motor point and phrenic nerve stimulation showed a significant difference, with much less amperage required at the phrenic nerve to initiate diaphragmatic contraction. An additional comparison with stimulation of the peripheral diaphragm muscle was excluded, as only a local contraction was visually detectable. On average, direct phrenic nerve stimulation resulted in diaphragmatic contraction at 0.26 ± 0.02 mA, compared with the significantly higher amperage of 7 ± 1.22 mA being required at the motor point (*p* = 0.005).

## Discussion

The current results show that for diaphragmatic contraction in the porcine model, stimulation directly at the phrenic nerve requires less amperage compared with the motor point and peripheral diaphragmatic muscle stimulation via a thoracic approach. Even with high amperages, stimulation of the peripheral diaphragm leads to inefficient contraction. Hence, direct phrenic nerve stimulation provides the most efficient pacing site, especially in the context of developing a fully implantable system to support a patient over the long term with a reduced need for battery changes. These results accord to the known studies on humans [1, 22] and mongrel dogs [24]. This suggests that our prospective device of a unilateral diaphragmatic pacemaker in porcine model is transferable to human patients in terms of the best target for stimulation.

Stimulation of intact versus transected nerves did not result in any relevant differences in amperage required. No significant change in pO2, pCO2, pH, respiratory minute volume, respiratory rate and diaphragmatic excursion was observed between the intact and transected phrenic nerve, independent of the location of stimulation. So, during phrenic nerve stimulation the effect of short-term phrenic nerve transection on all observed parameters appears to be minute. The acute loss of central nervous control therefore does not seem to have any significance for the amperage level required. In the setting of congenital cardiac surgery, clinically relevant unilateral diaphragmatic paresis usually occurs intraoperatively and can be treated promptly by diaphragmatic pacemaker implantation. In further studies, we plan to investigate how long a damaged phrenic nerve can still be effectively stimulated and how early phrenic nerve stimulation affects prevention of nerve dystrophy after nerve trauma.

In this study, the most commonly used stimulation locations were compared for the first time in the same setting [4, 24, 27]. In agreement with Bilgutay et al., we demonstrated the superiority of direct phrenic nerve stimulation compared with diaphragmatic pacing via the motor point or the even worse-performing peripheral diaphragmatic site. These data support diaphragmatic pacing via direct phrenic nerve stimulation as the most effective site, especially in light of Johnson et al.’s findings. They demonstrated increased tidal volume during diaphragmatic pacing compared with baseline at 6 weeks after the initial phrenic nerve injury [28]. Further data on the longevity of direct phrenic nerve stimulation after phrenic nerve trauma or damage are needed.

In our model, we opted for transthoracic instrumentation because of the intended use of the unilateral diaphragmatic pacemaker in children after cardiac surgery. A phrenic nerve injury would be spotted immediately after extubation, and in case of severe respiratory compromise, the implantation of a unilateral pacemaker could be performed using the existing sternotomy to minimize further surgical trauma. Additionally, direct phrenic nerve instrumentation provides the best results in terms of a low threshold required for diaphragmatic stimulation. In contrast, the abdominal approach carries the risk of further surgical trauma and allows only for electrode implantation at the motor point, resulting in higher required amperages [1, 22]. Additionally, a time-consuming mapping procedure must be performed [20]. In the current era, though, abdominal electrodes can be placed laparoscopically at the motor point, minimizing surgical trauma compared with conventional techniques [29].

Although augmented ventilation after stimulation of the phrenic nerve and the motor point was achieved, only the pCO_2_ significantly decreased. In addition, the pO_2_ in these two experimental groups showed only a trend towards an increase, and pH remained stable throughout the entire experiment. These findings are in accord with previously published results [30, 31].

### Limitations

This study was performed using healthy animals with normal organ function and without any previous operations. In particular,respiratory function was completely normal before the investigation. Thus, our results should be carefully interpreted, and direct extrapolation into the clinical setting is not warranted. Furthermore, the time frame in this study was brief, and no conclusions about longer term effects are possible. The experimental animals were ventilated during the entire experiment, including the period of diaphragmatic pacing, during which the level of ventilatory support was reduced to the absolute minimal level.

## Conclusion

Direct stimulation of the phrenic nerve seems also to be the best choice for stimulation in a porcine model by a unilateral thoracic diaphragm pacemaker in the context of the amperage level needed.

## Data Availability

The datasets used and/or analyzed during the current study are available from the corresponding author on reasonable request.
